# Comparison of survival outcomes between axillary conservation and axillary lymph node dissections in *N*1 early breast cancer: a propensity-matched SEER analysis

**DOI:** 10.1007/s12094-022-03017-0

**Published:** 2022-12-14

**Authors:** Nisha Wu, Xiaohan Su, Qiao Tan, Jing Luo, Yewei Yuan, Lingmi Hou, Junyan Li

**Affiliations:** 1 Academician (Expert) Workstation, Sichuan Key Laboratory of Medical Imaging, Department of Biological Targeting Laboratory of Breast Cancer, Breast and Thyroid Surgery, Affiliated Hospital of North Sichuan Medical College, 1# Maoyuan Road South, Shunqing District, Nanchong, 637000 Sichuan China; 2 Department of Clinical Laboratory, The Fifth Affiliated Hospital, Southern Medical University, Guangzhou, Guangdong China; 3 Department of Breast Surgery, Sichuan Provincial Maternity and Child Health Care Hospital, No. 290 West Second Street, Shayan Road, Chengdu, 610031 Sichuan China; 4 Department of General Surgery, Yingshan Hospital, Southwest Hospital of Sichuan University, Nanchong, Sichuan China

**Keywords:** Breast cancer, Sentinel lymph node dissection, Axillary lymph node dissection, Survival outcome, SEER database, Mastectomy

## Abstract

**Background:**

Sentinel lymph node dissection (SLND) is an alternative to axillary lymph node dissection (ALND) for breast cancer surgery. But the criteria of SLND only for patients with limited disease in the sentinel node is disputed.

**Methods:**

From the Surveillance, Epidemiology, and End Results (SEER) database, 2000–2015, we identified 97,296 early breast cancer females with 1–3 axillary lymph nodes macro-metastasis. Of them, 1–5 (axillary conservation group), 6–9, and ≥ 10 (ALND group) axillary lymph nodes were dissected in 28,639, 16,838, and 51,819 patients, respectively. According to the criteria of the ACOSOG Z0011 trial, two historical cohort studies of patients who underwent lumpectomy or mastectomy were conducted and the survival outcomes between ALND and axillary conservation were compared.

**Results:**

Overall, dissection of 6–9 regional lymph nodes resulted in the worst prognosis. After propensity-matched analysis, it was found that patients in the axillary conservation group had worse survival than the ALND group in overall survival. No significant difference in prognosis between the group undergoing lumpectomy was found both in OS and BCSS. Subgroup analysis revealed that Grade 3, *T*2, two lymph nodes positive, or Her2 positive were the main causes of worse survival in the axillary conservation group.

**Conclusion:**

Not all patients with *N*1 early breast cancer suit axillary conservation. Axillary conservation was sufficient in patients who were treated with lumpectomy. ALND cannot be omitted in patients who were ineligible for the Z0011 and undergoing mastectomy with the following characteristics: *T*2, Grade 3, two positive lymph nodes, and Her2 positive, which may be better complemented to the Z0011 trial. Hence, under different surgical methods, the clinical precision treatment of ALND or axillary preservation is essential.

**Supplementary Information:**

The online version contains supplementary material available at 10.1007/s12094-022-03017-0.

## Introduction

Axillary lymph node dissection (ALND) has been the standard approach to treat breast cancer for more than 100 years because of its reliability in identifying nodal metastases and achieving regional control. However, ALND is associated with a significant risk of complications such as lymphedema, numbness, axillary web syndrome, and decreased range of motion of the upper extremity [1]. Sentinel lymph node biopsy (SLNB) was, therefore, developed to accurately stage the tumor-draining axillary nodes with lower morbidity than that with ALND [2]. SLND alone is the accepted management for patients in whom sentinel lymph nodes (SLN) are histologically free of tumor. Axillary lymph node dissection (ALND), long used to identify women with axillary nodal metastases, was replaced as a staging procedure by the less morbid sentinel lymph node dissection (SLND) in the era of ZOO11 [3].

Currently, for patients with limited disease in the sentinel node, SLN biopsy (SLNB) alone, SLNB and nodal radiotherapy, and neoadjuvant chemotherapy are alternatives with supportive evidence in the setting of node-positive disease, (ACOSOG Z0011 trial, IBCSG 23-01 trial, and AMAROS trial) [4–6]. In addition, in patients with clinically node-positive breast cancer, neoadjuvant chemotherapy (NAC) can eradicate the disease in axillary lymph nodes, with nodal pathologic complete response rates exceeding 40%, reducing the need for axillary lymph node dissection (ALND) [7]. The ACOSOG Z0011 trial found that, among patients with *T*1–2 breast cancer, no palpable axillary lymph node, 1–2 sentinel lymph nodes containing metastases, and underwent lumpectomy and irradiation, overall survival of patients treated with sentinel lymph node dissection alone was noninferior to overall survival for those treated with axillary lymph node dissection [6]. The EORTC 10,981–22,023 AMAROS trial showed that for patients with *T*1–2 primary breast cancer, no palpable lymphadenopathy, and a positive sentinel node, axillary lymph node dissection, and axillary radiotherapy provide comparable axillary control [5].

According to the above researches, patients meeting the Z0011 criteria may be eligible to avoid ALND; however, a recommendation may sometimes be ambiguous for patients with positive SLNs who do not fulfill the Z0011 criteria [8]. Additionally, although studies have demonstrated that ALND can be replaced in certain conditions, subgroup analyses demonstrate that ALND produced a trend of better prognosis in some cases [5]. Even in the era of Z0011, a number of surgeons choose to perform ALND in patients who meet the criteria for axillary conservation. Therefore, even if multiple guidelines explicitly recommend axillary conservation, some patients undergo overtreatment. Evidence suggests that patients with potentially avoidable ALND represent 21% of the expected patients with lymphedema [9]. A recent survey revealed that 49.0% of surgeons (most of them were lower volume breast surgeons) recommended ALND for a single axillary lymph node with macro-metastasis [10]. In brief, since the Z0011 trial started the era of axillary conservation, to date, the debate over expanding or narrowing their indications has been continuing. This is primarily due to insufficient population-based real-world data.

To examine the practical effects of axillary conservation since the Z0011 trial, we reviewed the Surveillance, Epidemiology, and End Results (SEER) database of the US National Cancer Institute to compare the survival outcomes between ALND and axillary conservation in *N*1 early breast cancer to further explore the criteria of ALND alternatives.

## Materials and methods

### Data source

The SEER program includes the cancer incidence and mortality data of 18 population‐based registries that represent approximately 30% of the American population. We obtained the data from the SEER database using SEER*Stat software v8.3.6 based on November 2018 submission (1975–2016 varying).

### Patient selection

From 2001 to 2015, we identified 146,374 females with *T*1-2N1M0 breast cancer based on Breast-Adjusted AJCC 6th Stage. The following were the other selection criteria: axillary lymph nodes examined ≥ 1 and with 1–3 axillary lymph nodes macro-metastasis. The exclusion criteria were as follows: (1) age at diagnosis < 18 years or > 85 years; (2) diagnosis not confirmed; (3) patients with incomplete survival data and follow‐up information; (4) patients who did not undergo surgery; and (5) more than one malignancy. Finally, 97,296 patients were included in the study.

In the SEER database, the information regarding SLND and ALND is imperfect. Therefore, the number of regional nodes examined was used to determine the axillary lymph node surgical process [11]: 1–5 nodes examined represented axillary conservation surgery; ≥ 10 nodes examined represented ALND; 6–9 nodes examined represented uncertain procedures. Accordingly, all patients were categorized into the following three groups: Group 1 included patients in whom 1–5 regional nodes were examined; Group 2 included those in whom 6–9 regional nodes were examined; Group 3 included those in whom ≥ 10 regional nodes were examined.

### Study variables

Our primary outcome of interest was survival. Overall survival (OS) and breast cancer-specific survival (BCSS) were calculated from the date of diagnosis until the last date of available vital status. We also evaluated independent demographic and clinicopathological variables for each patient, including the age, sex, year of diagnosis (2001–2010 and 2011–2015), histologic grade (Grades 1, 2, and 3), histologic type (ductal, lobular, and ductal and lobular carcinoma, and others), T stage (Breast-Adjusted AJCC 6th edition T), hormone receptor status (ER-positive or PR-positive, ER-negative, and PR-negative), HER2/neu status, molecular subtype (Her2−/HR+, Her2+/HR+, Her2+/HR−, and triple-negative), type of surgery (partial mastectomy and mastectomy), number of axillary nodes positive (1, 2, and 3), radiotherapy, and chemotherapy.

### Statistical analysis

For demographic and clinicopathological data, continuous variables were compared using Student’s *t* test or analysis of variance (ANOVA), and categorical variables were compared using Pearson’s Chi-square test or rank-sum test. Survival curves were plotted according to the Kaplan–Meier method and compared using the log-rank test. Univariate and multivariate Cox’s proportional hazards regression models were constructed to analyze the factors associated with survival.

Subsequently, we performed propensity score matching (PSM) to further evaluate the effects of the number of regional nodes examined (1–5 or ≥ 10) on survival by adjusting for sex, year of diagnosis, histologic type, T stage, hormone receptor status, HER2 status, type of surgery, number of positive regional nodes, and radiation and chemotherapy (exact match and match tolerance = 0).

Lastly, according to the criteria of the ACOSOG Z0011 trial (women with clinical *T*1–*T*2 invasive breast cancer between 2001 and 2015 with 1–2 lymph nodes containing metastases), we also conducted two historical cohort studies of patients who underwent lumpectomy or mastectomy to evaluate the effects of the number of regional nodes examined on the survival.

Statistical significance was set at two‐sided *p* < 0.05 with 95% confidence intervals (CIs). Statistical analyses were performed using SPSS v25 (IBM Inc., Armonk, NY, USA) and Stata v16 (StataCorp LLC, College Station, TX, USA).

## Results

Overall, 97,296 females with early breast cancer with 1–3 axillary lymph nodes macro-metastasis were identified. Of them, Group 1 (1–5 lymph nodes were examined), Group 2 (6–9 lymph nodes were examined), and Group 3 (≥ 10 lymph nodes were examined) included 28,639, 16,838, and 51,819 patients, respectively. The demographic and clinicopathological characteristics of the three groups are summarized in Table 1. The rate of ALND decreased from 60.69 in 2001–2010 to 39.69% in 2011–2015, while the rate of axillary conservation increased from 21.15 to 44.56%. Compared to ALND, axillary were more likely to be conserved when early *N*1 breast cancer was *T*1 (58.35% vs. 48.02%, *p* < 0.001), hormone receptor positive (83.91% vs. 75.70%, *p* < 0.001), HER2 negative (49.03% vs. 26.57%, *p* < 0.001), or treated with partial mastectomy (49.03% vs. 26.57%, *p* < 0.001).

**Table 1:** Patient characteristics

Clinical characteristics	No. of patients (%)			*p*
	Group 1: 1–5 nodes examined, *n* = 28,639	Group 2: 6–9 nodes examined, *n* = 16,838	Group 3: ≥ 10 nodes examined, *n* = 51,819	
Age at diagnosis: mean ± SD, y	58.23 ± 12.575	56.62 ± 12.813	55.53 ± 12.429	< 0.001
Year of diagnosis				< 0.001
2001–2010	13,294 (21.15%)	11,417 (18.16%)	38,151 (60.69%)	
2011–2016	15,345 (44.56%)	5421 (15.74%)	13,668 (39.69%)	
Tumor grade				< 0.001
Unknown	942 (3.29%)	588 (3.49%)	1822 (3.52%)	
Grade I	5555 (19.40%)	2346 (13.93%)	6661 (12.85%)	
Grade II	13,346 (46.60%)	7410 (44.01%)	21,667 (41.81%)	
Grade III	8796 (30.72%)	6494 (38.56%)	21,669 (41.82%)	
Histologic type				< 0.001
Ductal carcinoma	21,933 (76.58%)	13,171 (78.22%)	40,574 (78.30%)	
Lobular carcinoma	2415 (8.43%)	1191 (7.07%)	3226 (6.23%)	
Ductal and lobular carcinoma	2266 (7.91%)	1302 (7.73%)	4162 (8.03%)	
Else type	2025 (7.07%)	1174 (6.97%)	3857 (7.44%)	
*T*				< 0.001
*T*0	31 (0.11%)	17 (0.10%)	103 (0.20%)	
*T*1	16,712 (58.35%)	8567 (50.88%)	24,882 (48.02%)	
*T*2	11,896 (41.54%)	8254 (49.02%)	26,834 (51.78%)	
Hormone receptor status				< 0.001
Unknown	1022 (3.57%)	824 (4.89%)	2453 (4.73%)	
Positive	24,030 (83.91%)	13,108 (77.85%)	39,227 (75.70%)	
Negative	3587 (12.52%)	2906 (17.26%)	10,139 (19.57%)	
HER2 status^a^				< 0.001
Unknown	12,438 (43.43%)	10,641 (63.20%)	35,000 (67.54%)	
Positive	2159 (7.54%)	1032 (6.13%)	3049 (5.88%)	
Negative	14,042 (49.03%)	5165 (30.67%)	13,770 (26.57%)	
Molecular subtype^a^				< 0.001
Unknown	12,459 (43.50%)	10,646 (63.23%)	35,030 (67.60%)	
HR+/Her2−	12,876 (44.96%)	4519 (26.84%)	11,776 (22.73%)	
HR+/Her2+	1623 (5.67%)	772 (4.58%)	2220 (4.28%)	
HR−/Her2+	534 (1.86%)	260 (1.54%)	819 (1.58%)	
HR−/Her2−	1147 (4.01%)	641 (3.81%)	1974 (3.81%)	
Surgery of breast				< 0.001
Unknown	29 (0.10%)	9 (0.05%)	41 (0.08%)	
Partial mastectomy	18,341 (64.04%)	8092 (48.06%)	22,948 (44.28%)	
Mastectomy	10,269 (35.86%)	8737 (51.89%)	28,830 (55.64%)	
Axillary nodes positive				
1	23,061 (80.52%)	10,139 (60.21%)	27,353 (52.79%)	
2	4413 (15.41%)	4478 (26.59%)	15,520 (29.95%)	
3	1165 (4.07%)	2221 (13.19%)	8946 (17.26%)	
Chemotherapy				< 0.001
Yes	16,516 (57.67%)	11,390 (67.64%)	37,331 (72.04%)	
No	12,123 (42.33%)	5448 (32.36%)	14,488 (27.96%)	
Radiotherapy				< 0.001
Yes	16,664 (58.19%)	8418 (49.99%)	24,499 (47.28%)	
No	11,975 (41.81%)	8420 (50.01%)	27,320 (52.72%)	

^a^the data was included in the SEER database since 2010

The demographic and clinicopathological characteristics of the three groups are summarized

**Fig. 1:**
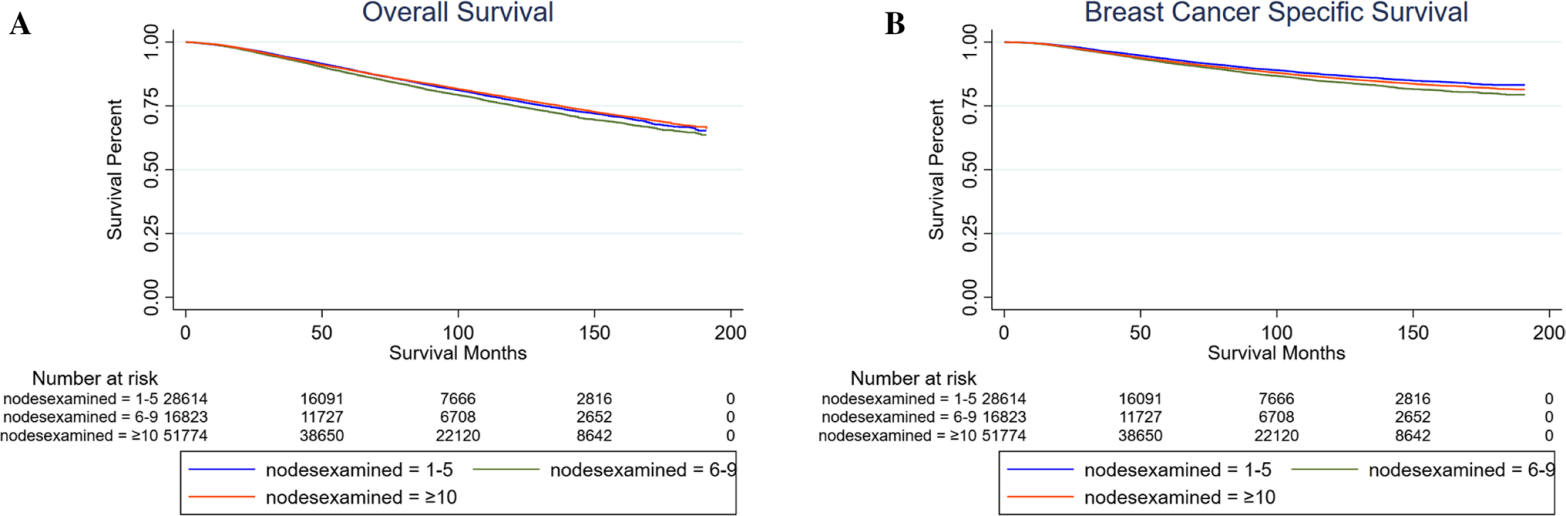
Kaplan–Meier curves of all *N*1 breast cancer patients (before PSM). **A** OS of the three groups. Patients in Group 2 (nodes examined = 6–9) had the worst survival (*p* < 0.001); no significant difference between Group 1 (nodes examined = 1–5) and Group 3 (nodes examined ≥ 10) (*p* = 0.3001). **B** BCSS of the three groups. Patients in Group 2 had the worst survival (*p* < 0.001). Patients in Group 1 had better prognosis than Group 3 (*p* < 0.001)

The median duration of follow-up was 56, 81, and 88 months for Groups 1, 2, and 3, respectively (*p* < 0.001). Kaplan–Meier curves that compare the survival between the three groups are shown in Fig. 1. Particularly, patients in Group 2 had the worst survival (*p* < 0.001). Between Group 1 and Group 3, there was no significant difference in the OS (*p* = 0.3001); however, patients in Group 1 had a better prognosis according to BCSS (*p* < 0.001). Multivariate Cox proportional hazards regression analyses revealed that the number of lymph nodes examined was an independent risk factor in terms of both OS and BCSS (Appendix, Supplementary Tables 1 and 2).

Propensity-matched analysis was performed between axillary conservation (1–5 lymph nodes examined) and ALND (≥ 10 lymph nodes examined).

**Fig. 2:**
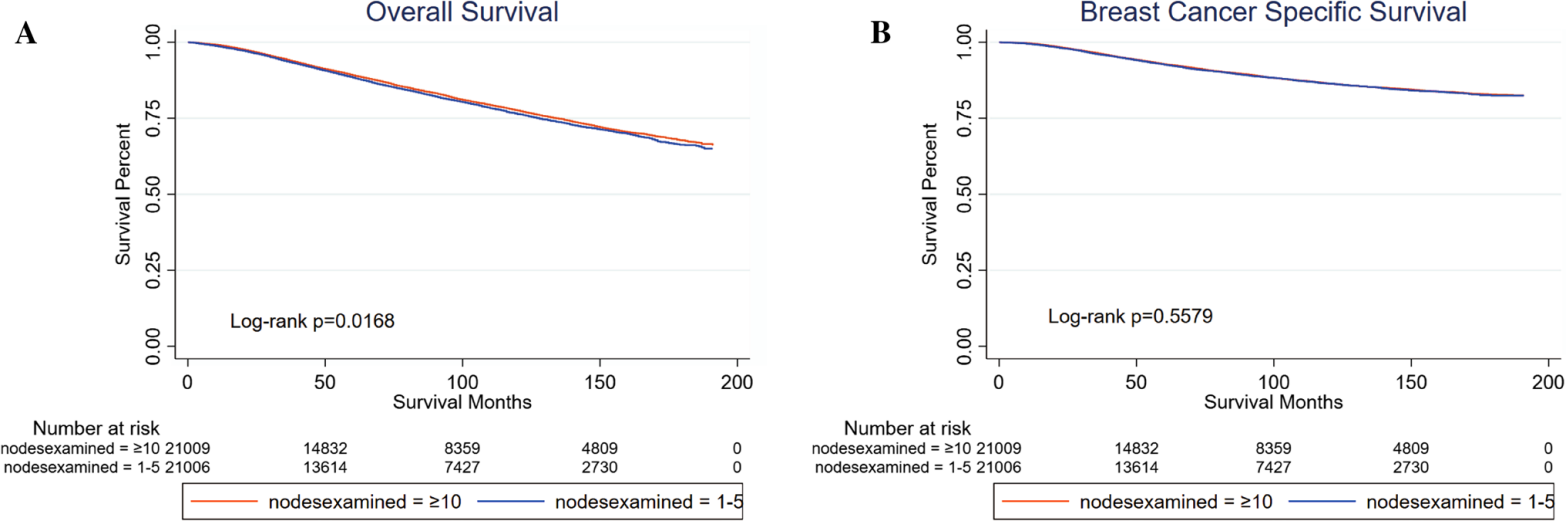
Kaplan–Meier curves of ALND and axillary conservation after PSM. **A** Overall survival. Patients in Group 1 (nodes examined = 1–5) had worse survival (*p* = 0.0168). **B** Breast cancer-specific survival. No significant difference between Group 1 (nodes examined = 1–5) and Group 3 (nodes examined ≥ 10) (*p* = 0.5579)

After PSM, 21,022 patients were included in each group and all the critical variables were balanced between them in Table 2. Kaplan–Meier curves comparing the survival between the two groups are shown in Fig. 2. Patients in Group 1 had worse survival in OS (*p* = 0.0168), while there was no significant difference between the two groups according to BCSS (*p* = 0.5579).

**Table 2:** Patient characteristics after PSM for patients with 1–5 or ≥ 10 lymph nodes examined

Clinical characteristics	No. of patients (%)		*p*
	Group 1: 1–5 nodes examined, *n* = 21,022	Group 3: ≥ 10 nodes examined, *n* = 21,022	
Age at diagnosis			1
≤ 50	6899 (32.82%)	6899 (32.82%)	
> 50	14,123 (67.18%)	14,123 (67.18%)	
Year of diagnosis			1
2001–2010	12,705 (60.44%)	12,705 (60.44%)	
2011–2016	8317 (39.56%)	8317 (39.56%)	
Tumor grade			1
Unknown	737 (3.51%)	737 (3.51%)	
Grade I	3687 (17.54%)	3687 (17.54%)	
Grade II	9544 (45.40%)	9544 (45.40%)	
Grade III	7054 (33.55%)	7054 (33.55%)	
Histologic type			1
Ductal carcinoma	16,402 (78.02%)	16,402 (78.02%)	
Lobular carcinoma	1548 (7.36%)	1548 (7.36%)	
Ductal and lobular carcinoma	1604 (7.63%)	1604 (7.63%)	
Else type	1468 (6.98%)	1468 (6.98%)	
*T*			1
*T*0	12 (0.06%)	12 (0.06%)	
*T*1	11,667 (55.50%)	11,667 (55.50%)	
*T*2	9343 (44.44%)	9343 (44.44%)	
Hormone receptor status			1
Unknown	869 (4.13%)	869 (4.13%)	
Positive	17,353 (82.55%)	17,353 (82.55%)	
Negative	2800 (13.32%)	2800 (13.32%)	
HER2 status^a^			1
Unknown	11,764 (55.96%)	11,764 (55.96%)	
Positive	1479 (7.04%)	1479 (7.04%)	
Negative	7779 (37.00%)	7779 (37.00%)	
Molecular subtype^a^			1
Unknown	11,766 (55.97%)	11,766 (55.97%)	
HR+/Her2−	6927 (32.95%)	6927 (32.95%)	
HR+/Her2+	1119 (5.32%)	1119 (5.32%)	
HR−/Her2+	360 (1.71%)	360 (1.71%)	
HR−/Her2−	850 (4.04%)	850 (4.04%)	
Surgery of breast			1
Unknown	5 (0.02%)	5 (0.02%)	
Partial mastectomy	11,448 (54.46%)	11,448 (54.46%)	
Mastectomy	9569 (45.52%)	9569 (45.52%)	
Nodes positive			1
1	15,951 (75.88%)	15,951 (75.88%)	
2	3970 (18.88%)	3970 (18.88%)	
3	1101 (5.24%)	1101 (5.24%)	
Chemotherapy			1
Yes	13,359 (63.55%)	13,359 (63.55%)	
No	7663 (36.45%)	7663 (36.45%)	
Radiotherapy			1
Yes	10,903 (51.86%)	10,903 (51.86%)	
No	10,119 (48.14%)	10,119 (48.14%)	

^a^the data was included in the SEER database since 2010

Propensity-matched analysis between axillary conservation (1–5 lymph nodes examined) and ALND (≥ 10 lymph nodes examined) after balanced all the critical variables

Separately, for patients treated with lumpectomy, Kaplan–Meier curves demonstrated no significant difference between the two groups both in OS (*p* = 0.1141) and BCSS (*p* = 0.8608). For patients treated with mastectomy, OS was different (*p* = 0.0384) but BCSS was not (*p* = 0.2559) (Appendix, Supplementary Fig. 1).

### Historical cohort study of patients undergoing lumpectomy according to ACOSOG Z0011 trial

**Fig. 3:**
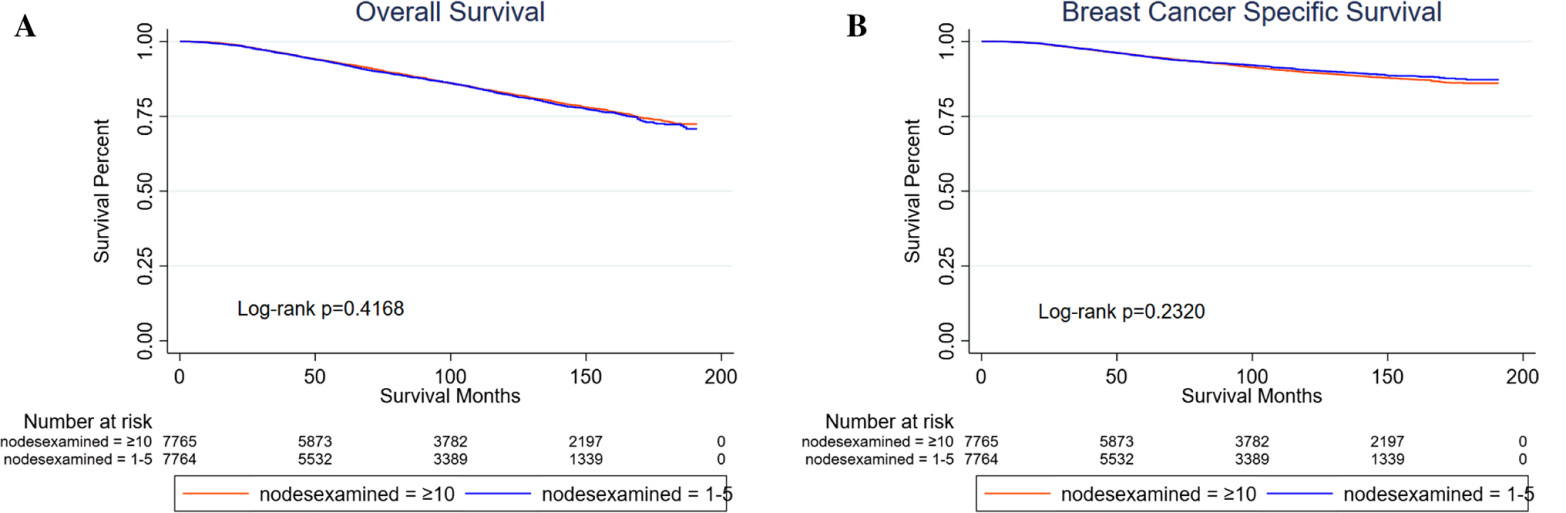
Kaplan–Meier curves of patients undergoing lumpectomy according to ACOSOG Z0011 trial. **A** Overall survival. No significant difference between Group 1 (nodes examined = 1–5) and Group 3 (nodes examined ≥ 10) (*p* = 0.417). **B** Breast cancer-specific survival. No significant difference between Group 1 and Group 3 (*p* = 0.232)

According to the inclusion criteria of ACOSOG Z0011 trial (women with clinical *T*1–*T*2 invasive breast cancer between 2000 and 2016 with 1–2 lymph nodes containing metastases who underwent lumpectomy and irradiation), a historical cohort study was performed between patients in whom 1–5 or ≥ 10 regional lymph nodes were dissected using PSM for critical clinicopathological characteristics (Appendix, Supplementary Table 3). Overall, 7,765 patients were included in each group and the survival results suggested that there was no significant difference both in OS (*p* = 0.4168) and in BCSS (95% CI *p* = 0.2320) (Fig. 3). In the subgroup analyses, no significant differences were observed in BCSS (Appendix, Supplementary Fig. 2).

### Historical cohort study of patients undergoing mastectomy similar to ACOSOG Z0011 trial

**Fig. 4:**
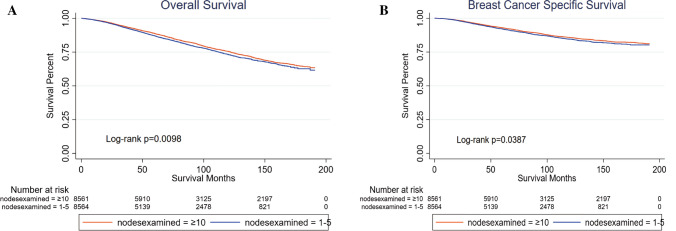
Kaplan–Meier curves of patients undergoing mastectomy similar to ACOSOG Z0011 trial. **A** Overall survival. Patients in whom 1–5 regional lymph nodes were dissected had worse prognosis (*p* = 0.0098). **B** Breast cancer-specific survival. Patients in whom 1–5 regional lymph nodes were dissected had worse prognosis (*p* = 0.0387)

To explore the effects of the number of dissected regional nodes on the survival in patients who underwent mastectomy, we conducted another historical cohort study of patients in whom 1–5 or ≥ 10 regional lymph nodes were dissected using PSM to identify the critical clinicopathological characteristics, referring to the inclusion criteria of the ACOSOG Z0011 trial (women with clinical *T*1–*T*2 invasive breast cancer between 2000 and 2016 with 1–2 lymph nodes containing metastases who underwent mastectomy); subsequently, 8,569 patients were included in each group (Appendix, Supplementary Table 4). Kaplan–Meier curves comparing survival in the three groups are shown in Fig. 4. Patients in whom 1–5 regional lymph nodes were dissected had a worse prognosis in both OS (*p* = 0.0098) and BCSS (*p* = 0.0387).

**Fig. 5:**
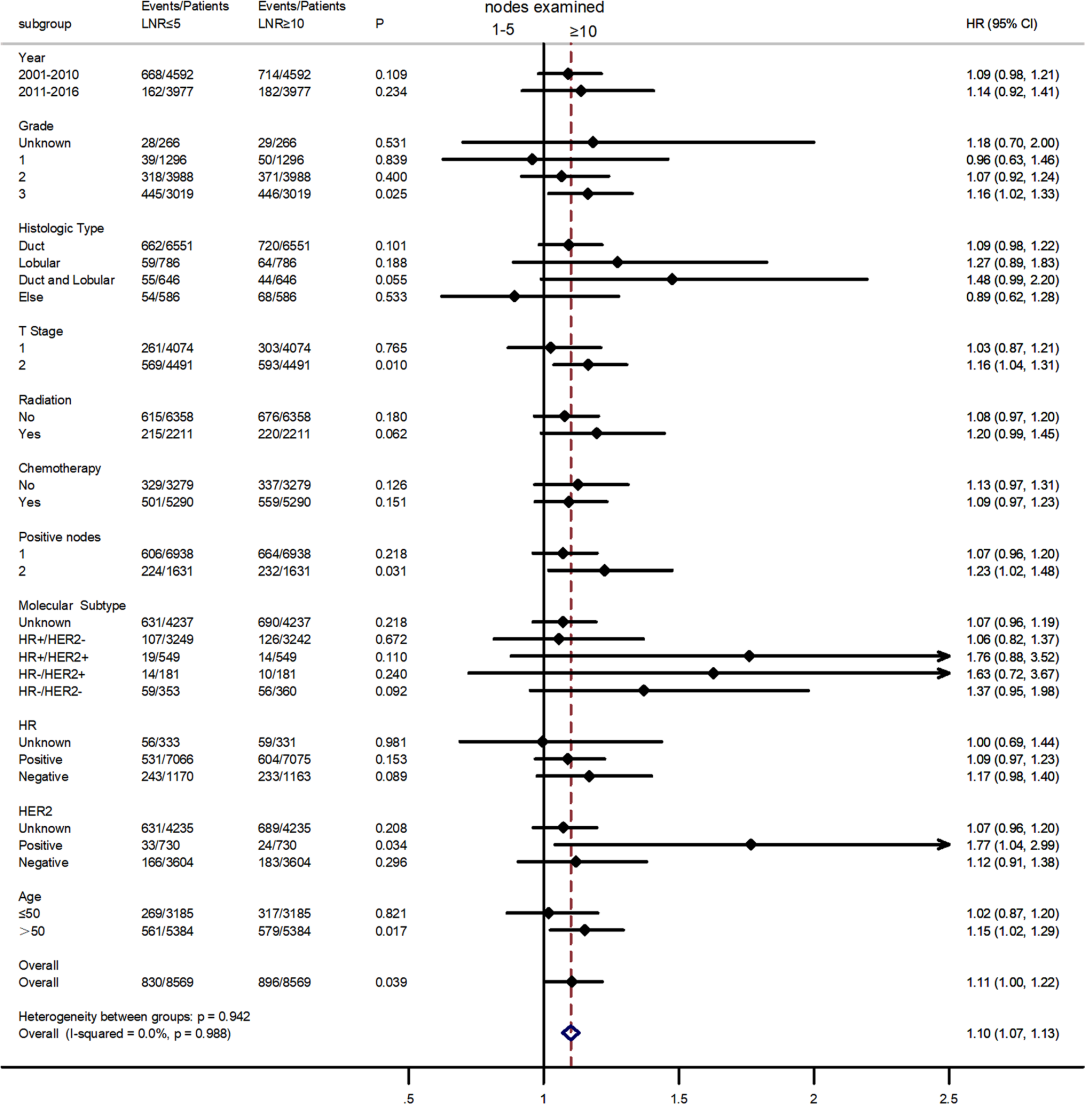
Subgroup analyses of BCSS for patients who underwent mastectomy similar to ACOSOG Z0011 trial. Under a few special conditions (histologic grade = 3; *T* stage = 2; regional nodes positive = 2; or Her2 status = positive), patients in whom 1–5 regional lymph nodes were dissected had worse prognosis

**Fig. 6:**
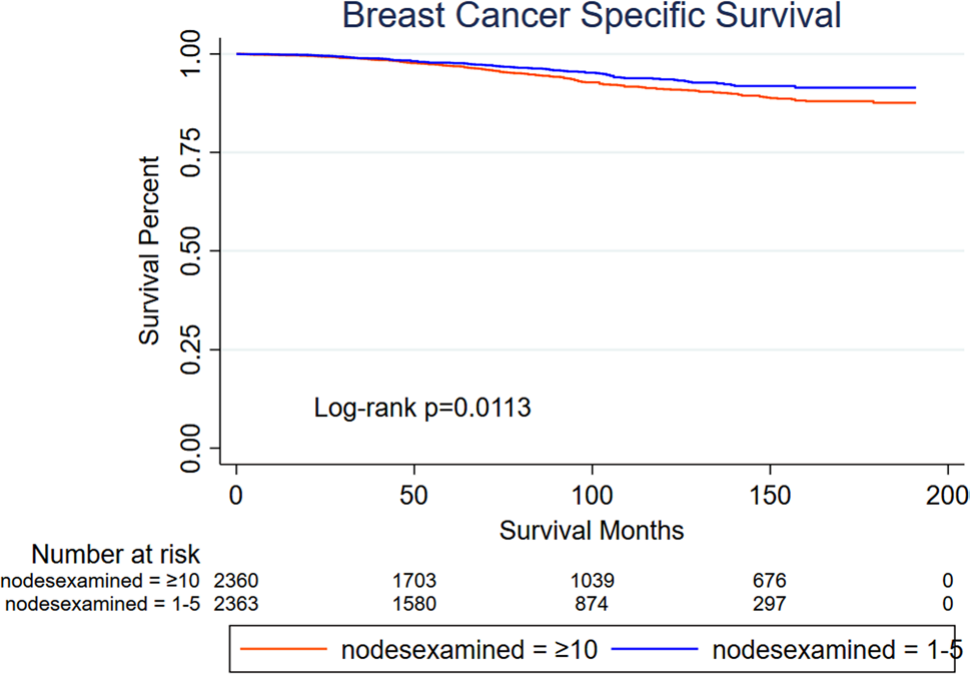
Kaplan–Meier curves of patients underwent mastectomy (*T*1, Grade 1/2, one positive lymph node, Her2 not positive). Axillary conservation (1–5 lymph nodes examined) resulted in a better prognosis (*p* = 0.0113)

The subsequent subgroup analyses suggested that, under a few special conditions (histologic grade = 3; *T* stage = 2; regional nodes positive = 2; or Her2 status = positive), patients underwent mastectomy in whom 1–5 regional lymph nodes were dissected (axillary conservation) and had worse prognosis in BCSS (Fig. 5). Surprisingly, when we excluded these high-risk factors, axillary conservation resulted in a better prognosis (*p* = 0.0113, Fig. 6). In addition, for patients treated with mastectomy, radiotherapy could not reduce the gap of prognosis between axillary conservation and ALND (Appendix, Supplementary Fig. 3).

## Discussion

In this study, we focused on the effects of axillary conservation or ALND on the prognosis of early-stage breast cancer. By reviewing the SEER database, we found that the rate of axillary conservation increased sharply since 2000. Generally, patients in axillary conservation group had worse survival than the ALND group in OS, while there was no significant difference in BCSS. Ji Hyeon Joo’s study also reported there was no difference in BCSS between ALND and no ALND in patients undergoing lumpectomy [3]. We further revealed that in patients undergoing breast-conserving surgery and radiotherapy who met the criteria of the Z0011 trial, axillary conservation, and ALND resulted in comparable survival outcomes. Moreover, our findings indicated that in patients who met the criteria of the Z0011 trial but were treated with mastectomy, the prognosis of those who underwent axillary conservation was significantly worse than the prognosis of those who underwent ALND, especially in the subgroups of those with Grade 3, *T*2, two lymph nodes positive, or Her2 positive. The results of AMAROS trial confirmed that the type of axillary management (axillary lymph node dissection) in patients with a positive sentinel node does not affect survival [5]. Even, the AMAROS trial suggests that for such patients, axillary radiotherapy is a valid treatment option with less morbidity than axillary lymph node dissection. The above examples further support our conclusion.

In the SEER database, the classification of the axillary surgery for breast cancer is inaccurate; therefore, we redefined axillary conservation as “dissection of 1–5 regional lymph nodes” and ALND as “dissection of ≥ 10 regional lymph nodes” [11]. Patients with 6–9 regional lymph nodes dissected could not be classified precisely. Our results demonstrate that their prognoses were worse than those in the other two groups. This finding confirms the validity of our classification of ALND and further suggests that reduction in the number of regional lymph nodes dissected is not a good approach for ALND alternatives [12]. However, we have to admit that the number of lymph nodes examined as an independent risk factor. First, the staging system for breast cancer follows the TNM system with the most recent system approved by the American Joint Committee. Second, biological factors may affect the predilection of some malignant cells to selectively invade lymph nodes first in breast cancer [13]. Third, clinically, the number of lymph nodes decided the surgical approach and clinical prognosis [14].

This study was a real-world retrospective analysis based on population data; therefore, the clinicopathological characteristics between the groups were not balanced. Preliminary survival analysis demonstrated that patients in whom 1–5 regional lymph nodes were dissected had the best BCSS outcome, which may have much to do with the surgeon’s tendency to select low-risk patients for axillary conservation. To reduce the selection bias, we performed a series of PSMs to adjust for critical clinicopathological characteristics. We chose 0 as match tolerance in each process, which assures identical critical clinicopathological characteristics between the two groups. After propensity-matched analysis, it was found that patients in the axillary conservation group had worse survival than the ALND group in OS, while there was no significant difference in BCSS.

Due to a significant risk of complications such as lymphedema, numbness, axillary web syndrome, and decreased range of motion of the upper extremity, finding an alternative treatment for ALND has always been one of the goals of breast cancer researchers [1]. Results from the ACOSOG Z0011 trial demonstrated that patients with limited disease in the sentinel node or nodes who are treated with breast-conserving surgery, whole-breast irradiation, and adjuvant systemic treatment can be spared from ALND without compromising the locoregional control or survival [6]. The Z0011 trial is of major importance in the management of patients with axillary lymph node-positive breast cancer [9]. Accordingly, an increasing number of patients who met the criteria of the Z0011 trial was averted from ALND [15]. Our results demonstrate that the rate of patients with *N*1 early breast cancer who underwent ALND decreased from 60.69 in 2001–2010 to 39.69% in 2011–2015, while the rate of axillary conservation increased from 21.15 to 44.56%. In contrast, in patients who were ineligible for the Z0011 trial, there were many attempts to avoid ALND [3, 16]. The Z0011 trial began an era of ALND alternatives for patients with limited disease in the sentinel node; however, some eligible patients still underwent axillary dissection [10]. In this study, a lot of patients who met the criteria of the Z0011 trial underwent ALND between 2011 and 2015.

Since the Z0011 trial, there were two contrasting attitudes toward axillary management. First, conservative surgeons demonstrated distrust of the trial, which resulted in overtreatment of eligible patients [10]. In this study, the analysis of a retrospective cohort simulating the Z0011 trial revealed that there was no significant difference in the prognosis of eligible patients who underwent axillary conservation or ALND. Furthermore, subgroup analysis did not reveal any special clinicopathological characteristics that could alter the results, which further excludes the potential risks [17, 18]. Second, radical surgeons wished to expand the indications for ALND alternatives so that more patients could be exempted from axillary dissection [14, 19]. As presented in our results, after propensity matched, patients in the axillary conservation group had worse survival than the ALND group in OS, while there was no significant difference in BCSS. The follow-up for patients with the 1–5 nodes group is limited. And the reasons were as follows: First, older adults accounted for the majority both in our study and in the Z0011trial, who suffered from many complications because of their poor general health physiological [20]. The adverse effects of surgery outweigh the clinical benefits in this subgroup. Therefore, it is necessary for us to explore precise treatment in early breast cancer surgical resection and axillary dissection.

For patients who were treated with mastectomy, our historical cohort study of patients who underwent mastectomy similar to the Z0011 trial demonstrated that the prognosis of the ALND group was still significantly better than the axillary conservation group, which differed from the results of some previous studies [3]. Subgroup analysis indicated that Grade 3, *T*2, two positive lymph nodes, and Her2 positive were the main reasons for the worse prognosis in the axillary conservation group. After eliminating these factors, axillary conservation even led to a better prognosis, which implied that in patients with *N*1 early breast cancer who undergo mastectomy, Grade 1/2, *T*1, one positive lymph node, and Her2 negative were indications for axillary conservation. The ongoing Senomac trial may provide more useful information for this aspect in the future. Previous studies showed that axillary radiotherapy is a very important alternative to ALND. Our results indicated that, for patients treated with mastectomy, radiotherapy could not reduce the gap of prognosis between axillary conservation and ALND. However, because there is no data on axillary radiotherapy in the SEER database, the significance of this finding is quite limited.

This study has several limitations. First, instead of SLND, we just compared the survival outcomes between axillary conservation and ALND in *N*1 early breast cancer, which may not fully reflect the real-world clinical practice. Second, “no palpable lymphadenopathy” is an essential criterion both in the ACOSOG Z0011 trial and the AMAROS trial. However, due to a lack of information in the SEER database, this study did not include this condition in the analysis. This study did not examine local–regional recurrence which was a major limitation because the SEER database does not contain data regarding recurrence. We suspect that these are the following reasons. First, the majority of women included in the SEER were older than 50 years or had hormone receptor positive disease, which caused subsequent follow-up information missed. Second, local–regional recurrence, defined as tumor recurrence in lymph nodes in the ipsilateral axilla, infraclavicular fossa, or interpectoral area, had to be confirmed with histological or fine needle examination [5]. Finally, there were no records of Her2 status and molecular subtype in the SEER database until 2010. An insufficient number of cases may lead to inaccuracy of the result.

## Conclusion

The safety of omitting ALND should be considered at all times, especially in patients with early-stage breast cancer. Therefore, we compare the survival outcomes between ALND and axillary conservation in *N*1 breast cancer patients undergoing lumpectomy or mastectomy in four historical cohort studies. In conclusion, not all patients with *N*1 early breast cancer are suitable for axillary conservation. Blindly expanding the indications for ALND alternatives will result in a poor prognosis. Nevertheless, axillary conservation is sufficient in patients who were ineligible for the Z0011 trial. Besides, although further related randomized trials are needed, patients with *T*1 breast cancer, Grade 1/2, one positive lymph node, Her2 negative, and undergoing mastectomy should be provided with axillary conservation rather than ALND.

## Supplementary Information

Below is the link to the electronic supplementary material.

**Table S1**: Multivariate Cox proportional hazards regression analyses of Overall Survival. Multivariate Cox proportional hazards regression analyses revealed that the number of lymph nodes examined was an independent risk factor in terms of OS. Grade (HR, 1.209; 95% CI 1.185–1.234; *p* < 0. 01), *T* stage (HR, 1.708; 95% CI 1.655–1.763; *p* < 0.01), Nodes-positive (HR, 1.194; 95% CI 1.170–1.219; *p* < 0. 01) or ER+ (HR, 1.371; 95% CI 1.328–1.415; *p* < 0.01) were independent risk factors. However, radiation (HR, 0.886; 95% CI 0.875–0.920; *p* < 0. 01), chemotherapy treatment (HR, 0.469; 95% CI 0.454–0.485; *p* < 0. 01) or HER2+ (HR, 0.934; 95% CI 0.901–0.967; *p* < 0. 01) could improve the OS in patients. **Table S2**: Multivariate Cox proportional hazards regression analyses of Breast Cancer Specific Survival. Multivariate Cox proportional hazards regression analyses revealed that the number of lymph nodes examined was an independent risk factor in terms of BCSS. Grade (HR, 1.455; 95% CI 1.413–1.498; *p* < 0. 01), T stage (HR, 2.019; 95% CI 1.934–2.108; *p* < 0.01), nodes-positive (HR, 1.267; 95% CI 1.234–1.301; *p* < 0. 01) or ER+ (HR, 1.573; 95% CI 1.510–1.638; *P* < 0.01) were independent risk factors. However, radiation (HR, 0.874; 95% CI 0.835–0.914; *p* < 0. 01), or chemotherapy treatment (HR, 0.750; 95% CI 0.717–0.784; *p* < 0. 01) could improve the BCSS in patients. There were no statistically significant in patients with HER2+ (*p* = 0.07). **Table S3**: Patient Characteristics of Historical cohort study of patients who underwent lumpectomy according to ACOSOG Z0011 trial. For patients treated with lumpectomy, Kaplan–Meier curves demonstrated no significant difference between the two groups both in OS and BCSS. For patients treated with mastectomy, OS was different but BCSS was not. **Table S4**: Patient Characteristics of Historical cohort study of patients who underwent mastectomy similar to ACOSOG Z0011 trial. For patients undergoing mastectomy, Kaplan–Meier curves demonstrated that patients in whom 1–5 or ≥ 10 regional lymph nodes were dissected to identify the critical clinicopathological characteristics, referring to the inclusion criteria of the ACOSOG Z0011 trial. **Fig. S1**: Kaplan–Meier curves of patients according to breast surgery (after PSM). **A**, **B** For patients treated with lumpectomy, no significant difference was found between the two groups both in OS and BCSS. Patients treated with mastectomy have a better overall survival rate than those who did not (**C**). However, no significant difference was found between BCSS (**D**). **Fig. S2**: Subgroup analyses of BCSS for patients undergoing lumpectomy according to the ACOSOG Z0011 trial. According to the ACOSOG Z0011 trial, no significant difference was found between the group with axillary conservation surgery (1–5 nodes examined) and the group with axillary lymph node dissection (> 10 nodes examined). **Fig. S3**: Kaplan–Meier curves of patients treated with mastectomy and radiotherapy. The patients with axillary conservation (1–5 nodes examined) tended to result in a worse prognosis. But there were no statistically significant between groups. (DOCX 415 KB)

## Data Availability

National Cancer Institute. Surveillance, Epidemiology, and End Results (SEER) Program (www.seer.cancer.gov) SEER*Stat Database: Incidence—SEER 18 Regs Custom Data (with additional treatment fields), November 2018 Sub (1975‐2016 varying)—Linked to County Attributes—Total US, National Cancer Institute, DCCPS, Surveillance Research Program, released April 2019, based on the November 2018 submission. Available at: https://seer.cancer.gov/data/, Accessed November 19, 2019.
