# Supplementary material for: Comparison of survival outcomes between axillary conservation and axillary lymph node dissections in N1 early breast cancer: a propensity-matched SEER analysis
Source: Clin Transl Oncol. 2022 Dec 14;25(4):1091–101. doi: 10.1007/s12094-022-03017-0 (PMC10025184; doi:10.1007/s12094-022-03017-0)
Supplement: Supplementary file 1 — Table S1: Multivariate Cox proportional hazards regression analyses of Overall Survival. Multivariate Cox proportional hazards regression analyses revealed that the number of lymph nodes examined was an independent risk factor in terms of OS. Grade (HR, 1.209; 95% CI 1.185–1.234; p < 0. 01), T stage (HR, 1.708; 95% CI 1.655–1.763; p < 0.01), Nodes-positive (HR, 1.194; 95% CI 1.170–1.219; p < 0. 01) or ER+ (HR, 1.371; 95% CI 1.328–1.415; p < 0.01) were independent risk factors. However, radiation (HR, 0.886; 95% CI 0.875–0.920; p < 0. 01), chemotherapy treatment (HR, 0.469; 95% CI 0.454–0.485; p < 0. 01) or HER2+ (HR, 0.934; 95% CI 0.901–0.967; p < 0. 01) could improve the OS in patients. Table S2: Multivariate Cox proportional hazards regression analyses of Breast Cancer Specific Survival. Multivariate Cox proportional hazards regression analyses revealed that the number of lymph nodes examined was an independent risk factor in terms of BCSS. Grade (HR, 1.455; 95% CI 1.413–1.498; p < 0. 01), T stage (HR, 2.019; 95% CI 1.934–2.108; p < 0.01), nodes-positive (HR, 1.267; 95% CI 1.234–1.301; p < 0. 01) or ER+ (HR, 1.573; 95% CI 1.510–1.638; P < 0.01) were independent risk factors. However, radiation (HR, 0.874; 95% CI 0.835–0.914; p < 0. 01), or chemotherapy treatment (HR, 0.750; 95% CI 0.717–0.784; p < 0. 01) could improve the BCSS in patients. There were no statistically significant in patients with HER2+ (p = 0.07). Table S3: Patient Characteristics of Historical cohort study of patients who underwent lumpectomy according to ACOSOG Z0011 trial. For patients treated with lumpectomy, Kaplan–Meier curves demonstrated no significant difference between the two groups both in OS and BCSS. For patients treated with mastectomy, OS was different but BCSS was not. Table S4: Patient Characteristics of Historical cohort study of patients who underwent mastectomy similar to ACOSOG Z0011 trial. For patients undergoing mastectomy, Kaplan–Meier curves demonstrated that patients in w [file 12094_2022_3017_MOESM1_ESM.docx]

**Supplementary Material**

| **Supplementary Table 1.** **Multivariate Cox proportional hazards regression analyses of**  **Overall Survival** | | | | |
| --- | --- | --- | --- | --- |
| **Clinical characteristics** | ***P*** | **Hazard ratio** | **95% Confidence Interval** | |
| Nodes-examined | ＜0.01 | 0.906 | 0.889 | 0.923 |
| YearGroup | ＜0.01 | 0.885 | 0.820 | 0.954 |
| Grade | ＜0.01 | 1.209 | 1.185 | 1.234 |
| HistGroup | 0.02 | 0.981 | 0.965 | 0.997 |
| T | ＜0.01 | 1.708 | 1.655 | 1.763 |
| SurgPrimSite | ＜0.01 | 1.177 | 1.137 | 1.219 |
| Radiation | ＜0.01 | 0.886 | 0.857 | 0.920 |
| Chemo | ＜0.01 | 0.469 | 0.454 | 0.485 |
| Nodes-positive | ＜0.01 | 1.194 | 1.170 | 1.219 |
| ER | ＜0.01 | 1.371 | 1.328 | 1.415 |
| HER2 | ＜0.01 | 0.934 | 0.901 | 0.967 |

| **Supplementary Table 2.** **Multivariate Cox proportional hazards regression analyses of**  **Breast Cancer Specific Survival** | | | | |
| --- | --- | --- | --- | --- |
| **Clinical characteristics** | ***P*** | **Hazard ratio** | **95% Confidence Interval** | |
| Nodes-examined | ＜0.01 | 0.894 | 0.872 | 0.917 |
| YearGroup | ＜0.01 | 0.837 | 0.763 | 0.918 |
| Grade | ＜0.01 | 1.455 | 1.413 | 1.498 |
| HistGroup | ＜0.01 | 0.961 | 0.940 | 0.982 |
| T | ＜0.01 | 2.019 | 1.934 | 2.108 |
| SurgPrimSite | ＜0.01 | 1.136 | 1.086 | 1.188 |
| Radiation | ＜0.01 | 0.874 | 0.835 | 0.914 |
| Chemo | ＜0.01 | 0.750 | 0.717 | 0.784 |
| Nodes-positive | ＜0.01 | 1.267 | 1.234 | 1.301 |
| ER | ＜0.01 | 1.573 | 1.510 | 1.638 |
| HER2 | 0.07 | 0.960 | 0.919 | 1.003 |

| **Supplementary Table 3. Patient Characteristics of Historical cohort study of patients underwent lumpectomy according to ACOSOG Z0011 trial** | | | |
| --- | --- | --- | --- |
| **Clinical characteristics** | **No. of Patients (%)** | | ***p*** |
|  | Group1,  1-5 nodes examined,  n=7765 | Group3,  ≥10 nodes examined,  n=7765 |  |
| Age at diagnosis |  |  | 1 |
| ≤50 | 2366(30.5%) | 2366(30.5%) |  |
| >50 | 5399(69.5%) | 5399(69.5%) |  |
| Year of diagnosis |  |  | 1 |
| 2000-2010 | 5196(66.9%) | 5196(66.9%) |  |
| 2010-2016 | 2569(33.1%) | 2569(33.1%) |  |
| Tumor grade |  |  | 1 |
| Unknown | 179(2.3%) | 179(2.3%) |  |
| Grade I | 1504(19.4%) | 1504(19.4%) |  |
| Grade II | 3518(45.3%) | 3518(45.3%) |  |
| Grade III | 2564(33.0%) | 2564(33.0%) |  |
| Histologic type |  |  | 1 |
| ductal carcinoma | 6317(81.4%) | 6317(81.4%) |  |
| lobular carcinoma | 403(5.2%) | 403(5.2%) |  |
| ductal and lobular carcinoma | 544(7.0%) | 544(7.0%) |  |
| else type | 501(6.5%) | 501(6.5%) |  |
| T |  |  | 1 |
| T0 | 2(<0.1%) | 2(<0.1%) |  |
| T1 | 5063(65.2%) | 5063(65.2%) |  |
| T2 | 2700(34.8%) | 2700(34.8%) |  |
| Hormone receptor status |  |  | 1 |
| Unknown | 209(2.7%) | 209(2.7%) |  |
| Positive | 6522(84.0%) | 6522(84.0%) |  |
| Negative | 1034(13.3%) | 1034(13.3%) |  |
| HER2 status^a^ |  |  | 1 |
| Unknown | 4773(61.5%) | 4773(61.5%) |  |
| Positive | 430(5.5%) | 430(5.5%) |  |
| Negative | 2562(33.0%) | 2562(33.0%) |  |
| Molecular Subtype^a^ |  |  | 1 |
| Unknown | 4773(61.5%) | 4773(61.5%) |  |
| HR+/Her2- | 2274(29.3%) | 2274(29.3%) |  |
| HR+/Her2+ | 327(4.2%) | 327(4.2%) |  |
| HR-/Her2+ | 103(1.3%) | 103(1.3%) |  |
| HR-/Her2- | 288(3.7%) | 288(3.7%) |  |
| Surgery of breast |  |  | 1 |
| Partial mastectomy | 7765(100%) | 7765(100%) |  |
| Regional nodes positive |  |  | 1 |
| 1 | 6243(80.4%) | 6243(80.4%) |  |
| 2 | 1522(19.6%) | 1522(19.6%) |  |
| Chemotherapy |  |  | 1 |
| Yes | 5350(68.9%) | 5350(68.9%) |  |
| No | 2415(31.1%) | 2415(31.1%) |  |
| Radiotherapy |  |  | 1 |
| Yes | 7765(100%) | 7765(100%) |  |

| **Supplementary Table 4. Patient Characteristics of Historical cohort study of patients underwent mastectomy similar to the ACOSOG Z0011 trial** | | | |
| --- | --- | --- | --- |
| **Clinical characteristics** | **No. of Patients (%)** | | ***p*** |
|  | Group1,  1-5 nodes examined,  n=8569 | Group3,  ≥10 nodes examined,  n=8569 |  |
| Age at diagnosis |  |  | 1 |
| ≤50 | 3185(37.2%) | 3185(37.2%) |  |
| >50 | 5384(62.8%) | 5384(62.8%) |  |
| Year of diagnosis |  |  | 1 |
| 2000-2010 | 4592(53.6%) | 4592(53.6%) |  |
| 2010-2016 | 3977(46.4%) | 3977(46.4%) |  |
| Tumor grade |  |  | 1 |
| Unknown | 266(3.1%) | 266(3.1%) |  |
| Grade I | 1296(15.1%) | 1296(15.1%) |  |
| Grade II | 3988(46.5%) | 3988(46.5%) |  |
| Grade III | 3019(35.2%) | 3019(35.2%) |  |
| Histologic type |  |  | 1 |
| ductal carcinoma | 6551(76.4%) | 6551(76.4%) |  |
| lobular carcinoma | 786(9.2%) | 786(9.2%) |  |
| ductal and lobular carcinoma | 646(7.5%) | 646(7.5%) |  |
| else type | 586(6.8%) | 586(6.8%) |  |
| T |  |  | 1 |
| T0 | 4(<0.1%) | 4(<0.1%) |  |
| T1 | 4074(47.5%) | 4074(47.5%) |  |
| T2 | 4491(52.4%) | 4491(52.4%) |  |
| Hormone receptor status |  |  | 0.984 |
| Unknown | 333(3.9%) | 331(3.9%) |  |
| Positive | 7066(82.5%) | 7075(82.6%) |  |
| Negative | 1170(13.7%) | 1163(13.6%) |  |
| HER2 status^a^ |  |  | 1 |
| Unknown | 4235(49.4%) | 4235(49.4%) |  |
| Positive | 730(8.5%) | 730(8.5%) |  |
| Negative | 3604(42.1%) | 3604(42.1%) |  |
| Molecular Subtype^a^ |  |  | 0.999 |
| Unknown | 4237(49.4%) | 4237(49.4%) |  |
| HR+/Her2- | 3249(37.9%) | 3242(37.8%) |  |
| HR+/Her2+ | 549(6.4%) | 549(6.4%) |  |
| HR-/Her2+ | 181(2.1%) | 181(2.1%) |  |
| HR-/Her2- | 353(4.1%) | 360(4.2%) |  |
| Surgery of breast |  |  | 1 |
| Mastectomy | 8569(100%) | 8569(100%) |  |
| Regional nodes positive |  |  | 1 |
| 1 | 6938(81.0%) | 6938(81.0%) |  |
| 2 | 1631(19.0%) | 1631(19.0%) |  |
| Chemotherapy |  |  | 1 |
| Yes | 5290(61.7%) | 5290(61.7%) |  |
| No | 3279(38.3%) | 3279(38.3%) |  |
| Radiotherapy |  |  | 1 |
| Yes | 2211(25.8%) | 2211(25.8%) |  |
| No | 6358(74.2%) | 6358(74.2%) |  |

| **Supplementary Figure 1. Kaplan-Meier curves of patients according to breast surgery (after PSM)** | |
| --- | --- |
| **A** | **B** |
| 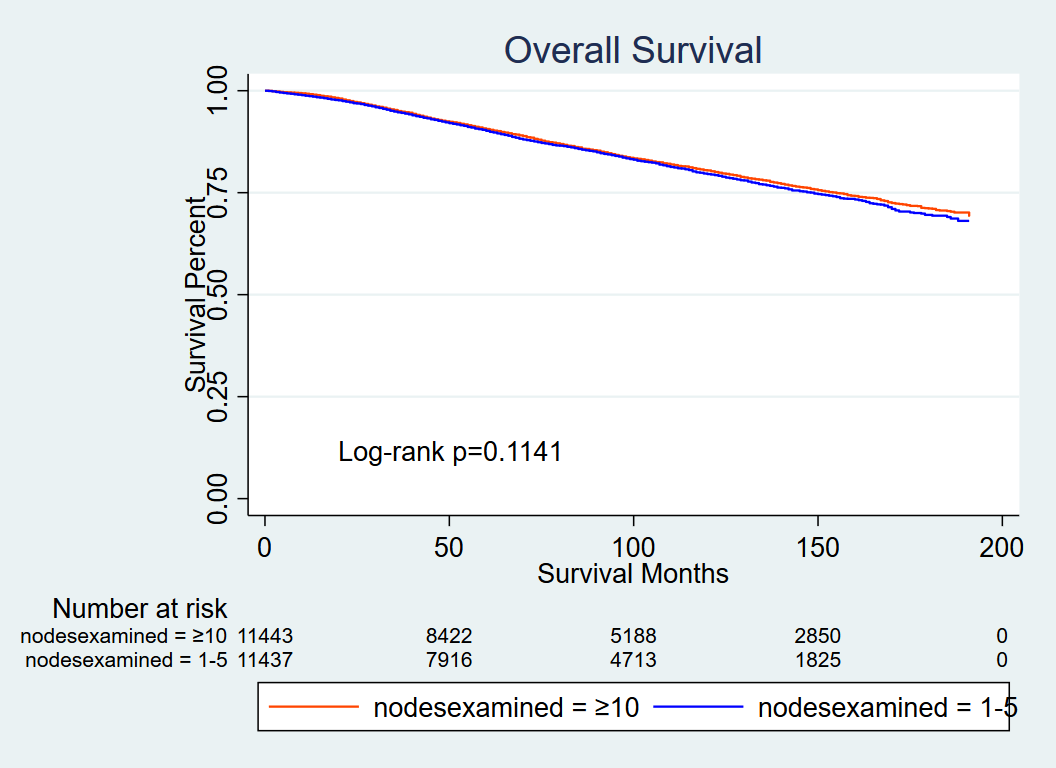 | 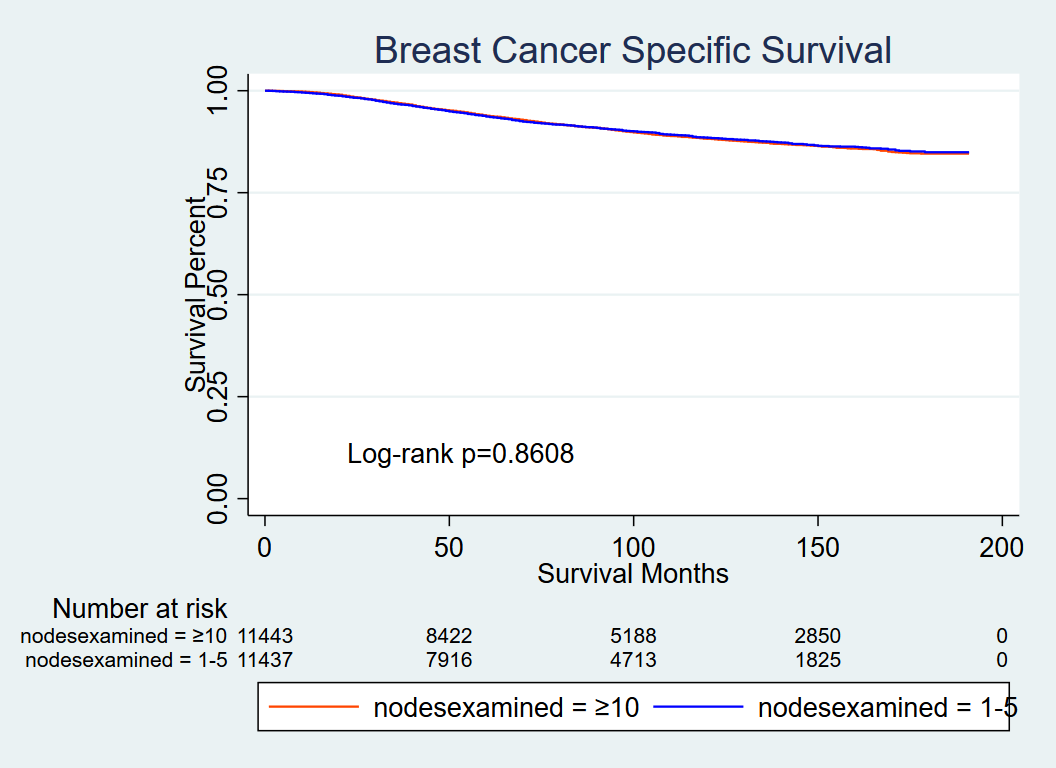 |
| **C** | **D** |
| 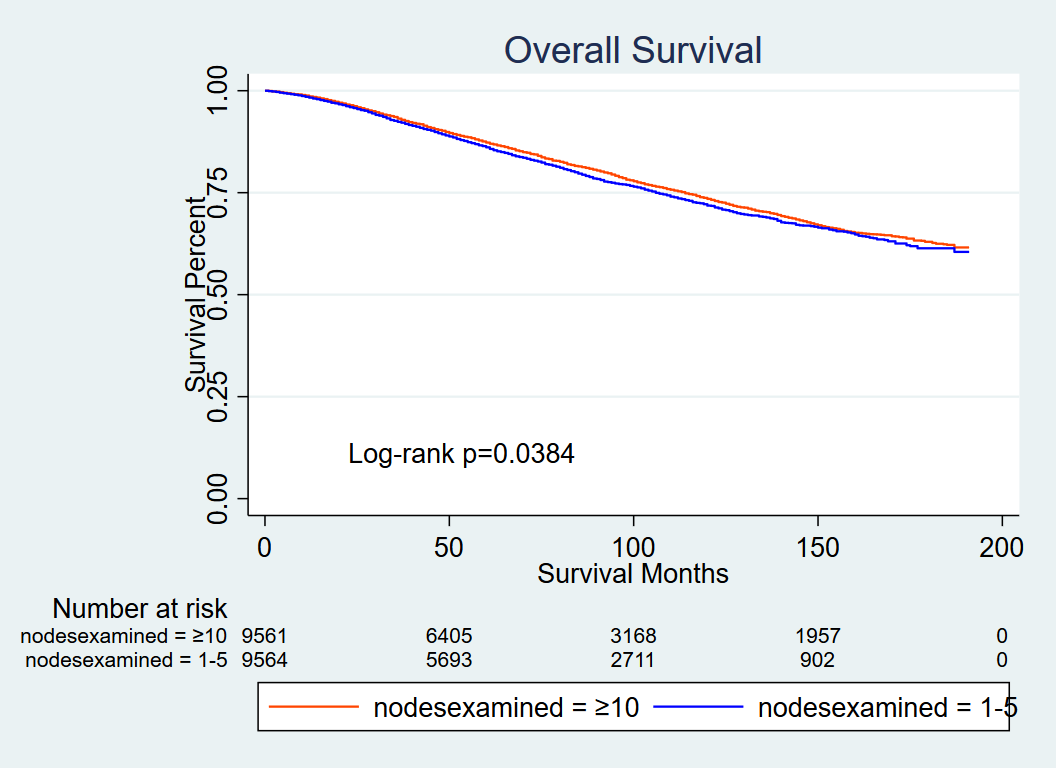 | 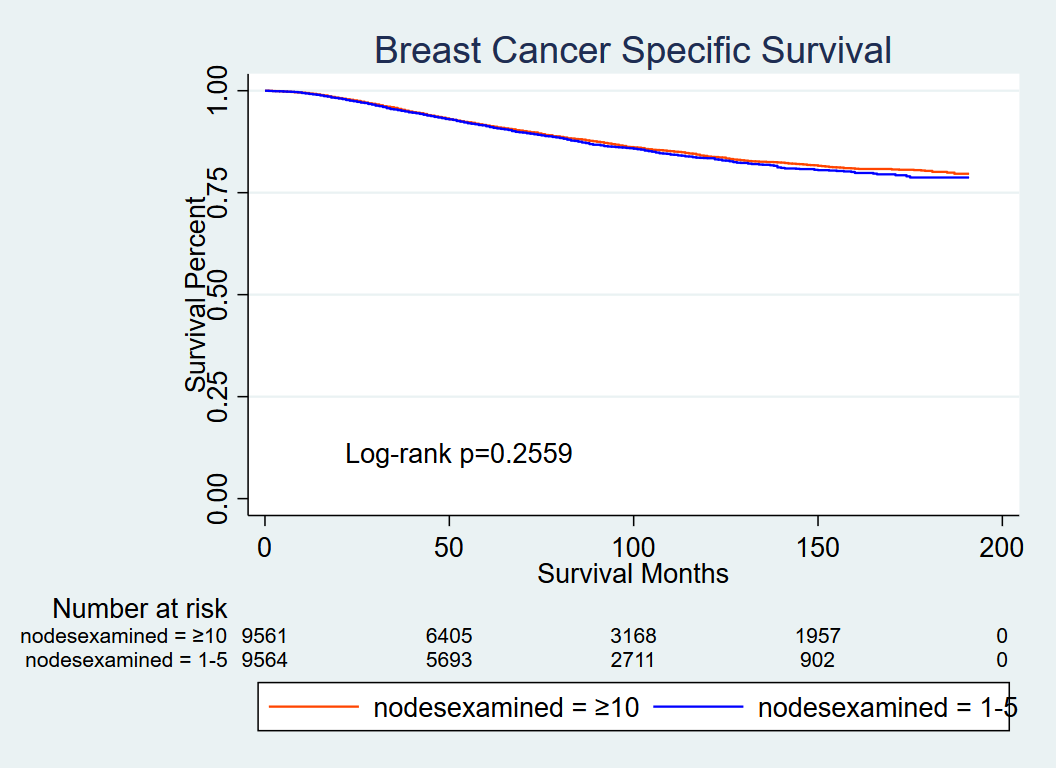 |

| **Supplementary Figure 2. Subgroup analyses of BCSS for patients undergoing lumpectomy according to the ACOSOG Z0011 trial.** 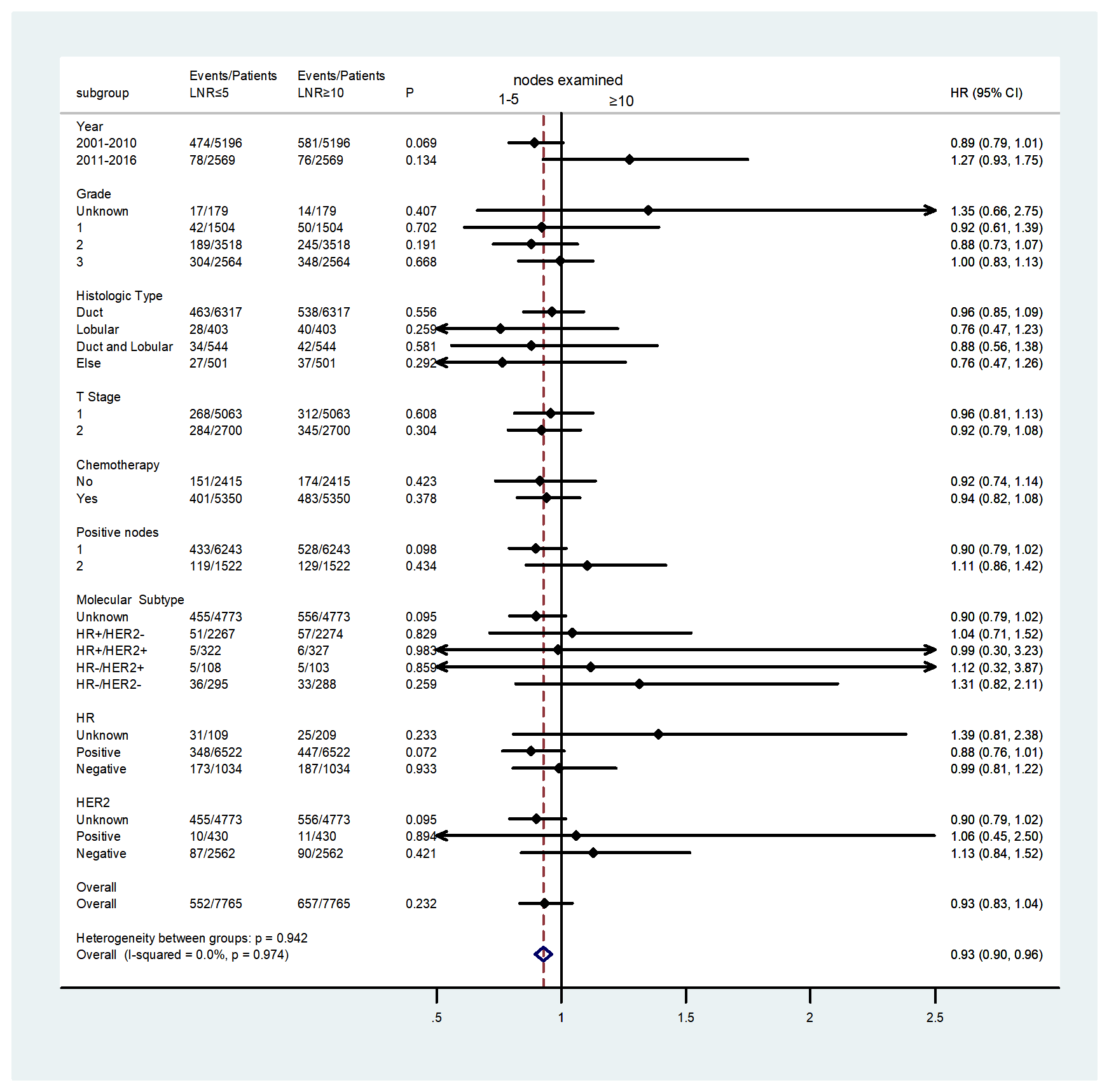 |
| --- |

| **Supplementary Figure 3. Kaplan-Meier curves of patients treated with mastectomy and radiotherapy.**  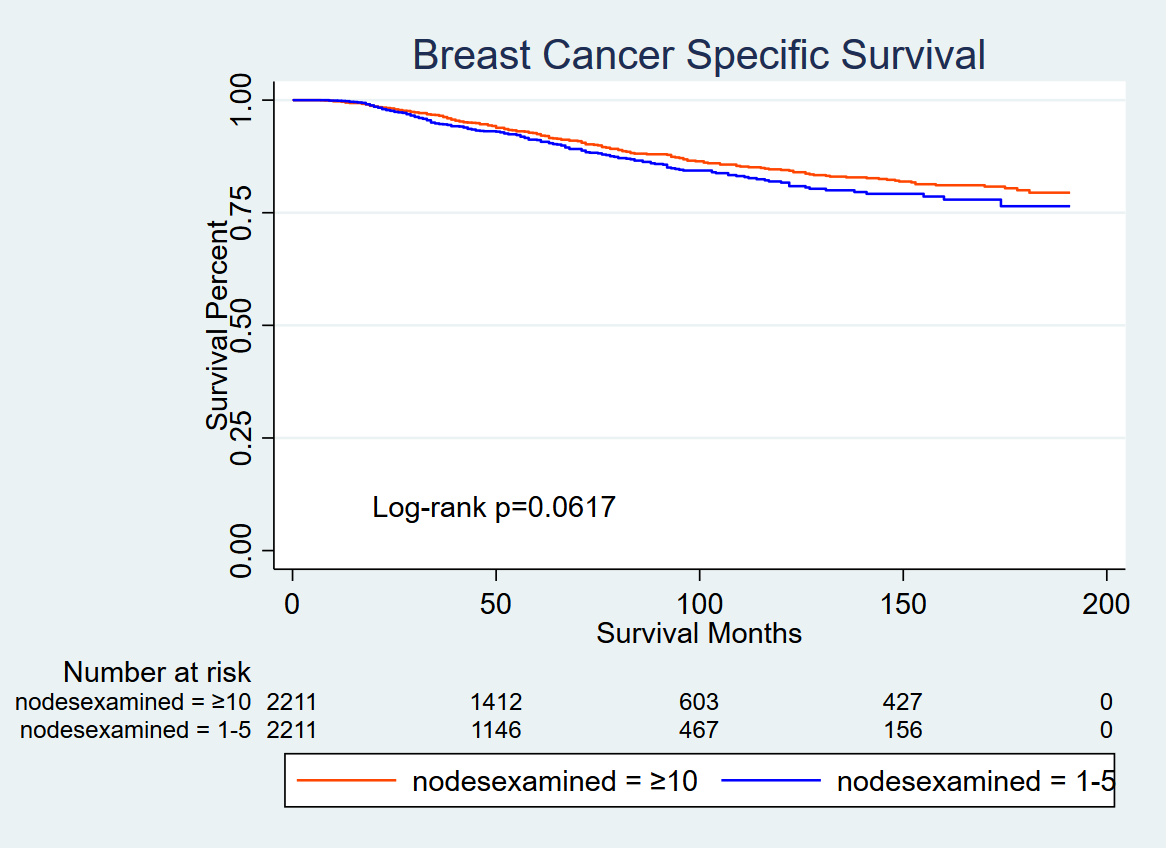 |
| --- |

**Supplementary Table 1.** **Multivariate Cox proportional hazards regression analyses of** **Overall Survival.**

Multivariate Cox proportional hazards regression analyses revealed that the number of lymph nodes examined was an independent risk factor in terms of OS. Grade (HR, 1.209; 95%CI, 1.185-1.234; *P*<0. 01), T stage (HR, 1.708; 95%CI, 1.655-1.763; *P*<0.01), Nodes-positive (HR, 1.194; 95%CI, 1.170-1.219; *P*<0. 01)or ER+ (HR, 1.371; 95%CI, 1.328-1.415; *P*＜0.01) were independent risk factors. However, radiation (HR, 0.886; 95%CI, 0.875-0.920; *P*<0. 01), chemotherapy treatment (HR, 0.469; 95%CI, 0.454-0.485; *P*<0. 01) or HER2+(HR,0.934; 95%CI, 0.901-0.967; *P*<0. 01) could improve the OS in patients.

**Supplementary Table 2. Multivariate Cox proportional hazards regression analyses of Breast Cancer Specific Survival.**

Multivariate Cox proportional hazards regression analyses revealed that the number of lymph nodes examined was an independent risk factor in terms of BCSS. Grade (HR, 1.455; 95%CI, 1.413-1.498; *P*<0. 01), T stage (HR, 2.019; 95%CI, 1.934-2.108; *P*<0.01), nodes-positive (HR, 1.267; 95%CI, 1.234-1.301; *P*<0. 01)or ER+ (HR, 1.573; 95%CI, 1.510-1.638; *P*＜0.01) were independent risk factors. However, radiation (HR, 0.874; 95%CI, 0.835-0.914; *P*<0. 01), or chemotherapy treatment (HR, 0.750; 95%CI, 0.717-0.784; *P*<0. 01) could improve the BCSS in patients. There were no statistically significant in patients with HER2+ (*P*=0.07).

**Supplementary Table 3. Patient Characteristics of Historical cohort study of patients who underwent lumpectomy according to ACOSOG Z0011 trial.**

For patients treated with lumpectomy, Kaplan–Meier curves demonstrated no significant difference between the two groups both in OS and BCSS. For patients treated with mastectomy, OS was different but BCSS was not.

**Supplementary Table 4. Patient Characteristics of Historical cohort study of patients who underwent mastectomy similar to ACOSOG Z0011 trial.**

For patients undergoing mastectomy, Kaplan–Meier curves demonstrated that patients in whom 1–5 or ≥10 regional lymph nodes were dissected to identify the critical clinicopathological characteristics, referring to the inclusion criteria of the ACOSOG Z0011 trial.

**Supplementary Figure 1. Kaplan-Meier curves of patients according to breast surgery (after PSM)**

(A), (B) For patients treated with lumpectomy, no significant difference was found between the two groups both in OS and BCSS. Patients treated with mastectomy have a better overall survival rate than those who did not (C). However, no significant difference was found between BCSS (D).

**Supplementary Figure 2. Subgroup analyses of BCSS for patients undergoing lumpectomy according to the ACOSOG Z0011 trial.**

According to the ACOSOG Z0011 trial, no significant difference was found between the group with axillary conservation surgery (1-5 nodes examined) and the group with axillary lymph node dissection (> 10 nodes examined).

**Supplementary Figure 3. Kaplan-Meier curves of patients treated with mastectomy and radiotherapy.**

The patients with axillary conservation (1-5 nodes examined) tended to result in a worse prognosis. But there were no statistically significant between groups. +
